# The effect of ventilator-associated pneumonia on the prognosis of intensive care unit patients within 90 days and 180 days

**DOI:** 10.1186/s12879-021-06383-2

**Published:** 2021-07-15

**Authors:** Wenjuan Luo, Rui Xing, Canmin Wang

**Affiliations:** Department of Critical Care Medicine, Guangdong Second Provincial General Hospital, No.466 Xingang Middle Road, Guangzhou, 510000 Guangdong China

**Keywords:** Ventilator-associated pneumonia, Prognosis, Intensive care unit

## Abstract

**Background:**

Mechanical ventilation (MV) is often applied in critically ill patients in intensive care unit (ICU) to protect the airway from aspiration, and supplement more oxygen. MV may result in ventilator-associated pneumonia (VAP) in ICU patients. This study was to estimate the 90-day and 180-day mortalities of ICU patients with VAP, and to explore the influence of VAP on the outcomes of ICU patients.

**Methods:**

Totally, 8182 patients who aged ≥18 years and received mechanical ventilation (MV) in ICU from Medical Information Mart for Intensive Care III (MIMIC III) database were involved in this study. All subjects were divided into the VAP group (*n* = 537) and the non-VAP group (*n* = 7626) based on the occurrence of VAP. Clinical data of all participants were collected. The effect of VAP on the prognosis of ICU patients was explored by binary logistic regression analysis.

**Results:**

The results delineated that the 90-day mortality of VAP patients in ICU was 33.33% and 180-day mortality was 37.62%. The 90-day and 180-day mortality rates were higher in the VAP group than in the non-VAP group. After adjusting the confounders including age, ethnicity, heart failure, septicemia, simplified acute physiology score II (SAPSII) score, sequential organ failure assessment (SOFA) score, serum lactate, white blood cell (WBC), length of ICU stay, length of hospital stay, length of ventilation, antibiotic treatment, *Pseudomonas aeruginosa* (P.aeruginosa), methicillin-resistant *Staphylococcus aureus* (MRSA), other pathogens, the risk of 90-day and 180-day mortalities in VAP patients were 1.465 times (OR = 1.465, 95%CI: 1.188–1.807, *P* < 0.001) and 1.635 times (OR = 1.635, 95%CI: 1.333–2.005, *P* < 0.001) higher than those in non-VAP patients, respectively.

**Conclusions:**

Our study revealed that ICU patients with VAP had poorer prognosis than those without VAP. The results of this study might offer a deeper insight into preventing the occurrence of VAP.

## Background

Mechanical ventilation (MV) is often applied in critically ill patients in intensive care unit (ICU) [1]. MV may result in various complications and bring substantial risks in ICU patients [2]. Ventilator-associated pneumonia (VAP) is a common nosocomial infection in ICU occurring > 48 h after endotracheal intubation in patients receiving MV [3]. The most common clinical symptoms of VAP patients were fever, changed white blood cell count, altered sputum characteristic, appearance of a causative agent [4]. VAP can be considered when new pulmonary infiltrates appear, and diseases such as pulmonary edema, pulmonary tumors, and pulmonary infarction have been excluded [5].

VAP is associated with high morbidity and mortality. Previous researchers have identified that the incidence of VAP is approximately 8 to 28% [6]. Although the tendency of incidence was decreased in recent years, VAP is still a heavy burden to the patients and society. The occurrence of VAP may lead to the prolongation of MV use, the consumption of antibiotics, and also increase of length of stay and the burden of hospitalization costs [7]. Multiple lines of evidences revealed that the prognosis of VAP patients is very poor [8]. The mortality of VAP was recorded to be 19.4–51.6% in China and 14 to 50% in other countries [9, 10]. Critically ill patients are always admitted to the ICU and present high mortality risk [11]. VAP in ICU has an incidence of 13.5 to 23%, and accounts for one of the common causes of morbidity and mortality [12, 13]. Currently, the effect of VAP on the prognosis of ICU patients was still not fully elucidated. A detailed understanding of VAP may have important implications for improving the outcomes of patients with VAP. The aim of this study was to estimate the 90-day and 180-day mortalities of ICU patients with VAP, and to explore the influence of VAP on the outcomes of ICU patients. The results of our study might provide a reference for clinicians to make timely intervention for prevent the occurrence of VAP in ICU.

## Methods

### Study population

Medical Information Mart for Intensive Care III (MIMIC III) database (https://mimic.physionet.org/) is a freely accessible database comprising the data associated with health of about 60,000 patients staying in the critical care units of Beth Israel Deaconess Medical Center from 2001 to 2012 [14]. In our study, the data about 8182 patients receiving MV in ICU was extracted from MIMIC III database. Those aged < 18 years were excluded (*n* = 19). Finally, 8163 subjects were involved. All subjects were divided into the VAP group (*n* = 537) and the non-VAP group (*n* = 7626) based on the occurrence of VAP. The diagnosis of VAP was in line with the criteria of the American Centers for Disease Control and Prevention (CDC) [15]. The CDC algorithm defines probable VAP include clinically nuanced, subjective criteria such as worsening gas exchange, change in the character of sputum, and new or progressive and persistent infiltrates (Table 1) [16]. The construction of MIMIC-III database was approved by the Ethics Review Board of the Beth Israel Deaconess Medical Center and all private information has been carried out the desensitization.

**Table 1:** Centers for Disease Control and Prevention’s clinical surveillance definition for VAP

Radiologic criteria (two or more serial radiographs with at least one of the following)	1. New or progressive and persistent infiltrate
	2. Consolidation
	3. Cavitation
Systemic criteria (at least one)	1. Fever (> 38 °C or > 100.4 °F)
	2. Leukopenia (< 4000 WBC/mm^3^) or leukocytosis (≥12,000 WBC/mm^3^)
	3. For adults ≥70 years old, altered mental status with no other recognized cause
Pulmonary criteria (at least two)	1. New onset of purulent sputum, or change in character of sputum, or increased respiratory secretions, or increased suctioning requirements
	2. Worsening gas exchange (e.g., desaturations, increased oxygen requirements, or increased ventilator demand
	3. New onset or worsening cough, or dyspnea, or tachypnea
	4. Rales or bronchial breath sounds

### Data extraction

Clinical data of all participants were collected from MIMIC III database based on the clinical experience and other literatures have published previously with a relevant topic. The data included age (years), gender, ethnicity (White, Asian, Black, Hispanic/latino and Other), red cell distribution width (RDW), white blood cell (WBC, 10^9^/L), international normalized ratio (INR), length of ICU stay (day), length of hospital stay (day), length of ventilation (hour), antibiotic treatment (none, single antibiotic, and combined antibiotics), pathogens species [Acinetobacter baumannii (A.baumannii), *Pseudomonas aeruginosa* (P.aeruginosa), methicillin-resistant *Staphylococcus aureus* (MRSA), *Klebsiella pneumoniae* (K.pneumoniae), methicillin-sensitive Staphylo coccus aureus (MSSA), *Escherichia coli* (*E. coli*), and Other pathogens], serum lactate (mmol/L), history of chronic obstructive pulmonary disease (COPD), diabetes, septicemia and heart failure, simplified acute physiology score II (SAPSII) score, sequential organ failure assessment (SOFA) score, 90-day mortality, and 180-day mortality.

### Statistical analysis

All statistical analyses were completed by R 4.0.2 software. Wilcoxon rank sum test was used for comparison of quantitative variables between the VAP group and non-VAP group, while chi-square test or Fisher’s exact test were used for comparing the qualitative variables. The effect of VAP on the prognosis of ICU patients was explored by binary logistic regression analysis with the occurrence of VAP as the independent variable and death within 90/180 days as the dependent variables after adjusting the confounders (age, ethnicity, heart failure, septicemia, SAPSII score, SOFA score, serum lactate, WBC, length of ICU stay, length of hospital stay, length of ventilation, antibiotic treatment, P.aeruginosa, MRSA, other pathogens). Two-side test was used in this study, and *P* < 0.05 was considered statistically significant.

## Results

### Clinical characteristics of all subjects

**Fig. 1:**
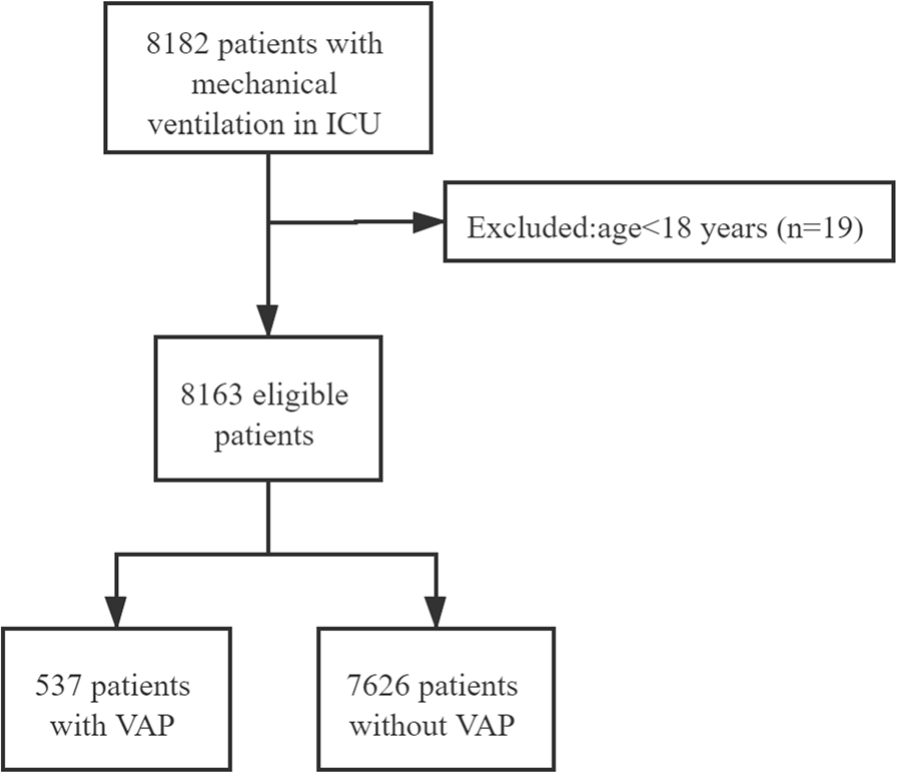
The screen process of all the subjects in our study

In total, 8182 patients receiving MV in ICU were involved in our study. After excluding 19 patients who aged < 18 years, 8136 subjects were involved in this study, including 537 (6.58%) in the VAP group and 7626 (93.42%) in the non-VAP group. The screening process of patients was shown in Fig. 1. Among all patients, the average age of all participants was 64.06 ± 16.28 years, and 4940 (60.52%) patients were males.215 (2.63%) people were Asians, 563 (6.90%) people were Black, 298 (3.65%) people were Hispanic/latino and 5902 (72.30%) people were White. 861 (10.55%) subjects had COPD, 1493 (18.29%) had septicemia, 2027 (24.83%) had and 2107 (25.81%) had heart failure. The 90-day and 180-day mortality rates were 24.61 and 26.60%, respectively (Table 2).

**Table 2:** Comparison and single logistic analysis for Characteristics of VAP and Non-VAP patients

Characteristic	All patients (*n* = 8163)	Non-VAP Patients (*n* = 7626)	VAP Patients (*n* = 537)	Statistical magnitude	*P*	OR (95%CI)	Logit*-P*
Age, Mean ± SD	64.06 ± 16.28	64.19 ± 16.22	62.29 ± 16.98	t = 2.620	0.009	0.993 (0.988–0.998)	0.009
Gender, n(%)				χ^2^ = 0.132	0.716		
Female	3223 (39.48)	3007 (39.43)	216 (40.22)			Ref	
Male	4940 (60.52)	4619 (60.57)	321 (59.78)			0.967 (0.809–1.156)	0.716
Ethnicity, n(%)				χ^2^ = 15.423	0.004		
White	5902 (72.30)	5543 (72.69)	359 (66.85)			Ref	
Asian	215 (2.63)	197 (2.58)	18 (3.35)			1.411 (0.861–2.313)	0.172
Black	563 (6.90)	515 (6.75)	48 (8.94)			1.439 (1.051–1.971)	0.023
Hispanic/latino	298 (3.65)	285 (3.74)	13 (2.42)			0.704 (0.400–1.240)	0.225
Other	1185 (14.52)	1086 (14.24)	99 (18.44)			1.408 (1.116–1.775)	0.004
COPD, n(%)	861 (10.55)	794 (10.41)	67 (12.48)	χ^2^ = 2.267	0.132	1.227 (0.940–1.601)	0.133
Heart failure, n(%)	2107 (25.81)	1935 (25.37)	172 (32.03)	χ^2^ = 11.607	< 0.001	1.386 (1.148–1.674)	< 0.001
Diabetes, n(%)	2027 (24.83)	1887 (24.74)	140 (26.07)	χ^2^ = 0.473	0.492	1.073 (0.878–1.309)	0.492
Septicemia, n(%)	1493 (18.29)	1324 (17.36)	169 (31.47)	χ^2^ = 66.828	< 0.001	2.186 (1.805–2.647)	< 0.001
SAPSII score, M(Q_1_,Q_3_)	38.00 (30.00,49.00)	38.00 (30.00,49.00)	41.00 (31.00,52.00)	Z = 3.679	< 0.001	1.009 (1.003–1.014)	0.002
SOFA score, Mean ± SD	7.128 ± 3.581	7.096 ± 3.579	7.581 ± 3.585	t = −3.040	0.002	1.037 (1.013–1.062)	0.002
Serum Lactate, M(Q_1_,Q_3_)	1.60 (1.10,2.60)	1.60 (1.10,2.60)	1.80 (1.20,3.00)	Z = 2.773	0.006	1.051 (1.011–1.092)	0.013
WBC, M(Q_1_,Q_3_)	11.60 (8.20,15.70)	11.50 (8.10,15.70)	12.00 (8.70,16.20)	Z = 2.068	0.039	1.010 (1.001–1.018)	0.022
INR, M(Q_1_,Q_3_)	1.30 (1.10,1.50)	1.30 (1.10,1.50)	1.20 (1.10,1.60)	Z = -1.295	0.195	1.067 (0.998–1.141)	0.059
Length of ICU stay, M(Q_1_,Q_3_)	3.17 (1.61,6.98)	2.99 (1.48,6.06)	12.69 (7.69,20.81)	Z = 27.865	< 0.001	1.140 (1.128–1.152)	< 0.001
Length of hospital stay, M(Q_1_,Q_3_)	8.52 (5.14,14.65)	8.09 (5.00,13.48)	18.79 (12.33,26.91)	Z = 21.875	< 0.001	1.046 (1.040–1.052)	< 0.001
Length of ventilation, M(Q_1_,Q_3_)	23.50 (8.63,87.33)	20.86 (7.85,69.67)	216.47 (114.57,351.26)	Z = 27.780	< 0.001	1.008 (1.007–1.008)	< 0.001
Antibiotic treatment, n(%)				Z = 18.883	< 0.001		
None	1266 (15.51)	1248 (16.37)	18 (3.35)			Ref	
Single antibiotic	2623 (32.13)	2610 (34.23)	13 (2.42)			0.346 (0.169–0.707)	0.004
Combined antibiotics	4274 (52.36)	3768 (49.41)	506 (94.23)			9.311 (5.793–14.963)	< 0.001
Pathogens species, n(%)							
A.baumannii	6 (0.07)	5 (0.07)	1 (0.19)	–	0.335	2.850 (0.333–24.400)	0.339
P.aeruginosa	67 (0.82)	53 (0.69)	14 (2.61)	–	< 0.001	3.825 (2.108–6.939)	< 0.001
MRSA	181 (2.22)	156 (2.05)	25 (4.66)	χ^2^ = 15.760	< 0.001	2.339 (1.519–3.602)	< 0.001
K.pneumoniae	61 (0.75)	54 (0.71)	7 (1.30)	–	0.120	1.852 (0.839–4.090)	0.127
MSSA	123 (1.51)	113 (1.48)	10 (1.86)	χ^2^ = 0.489	0.484	1.262 (0.657–2.423)	0.485
*E. coli*	128 (1.57)	118 (1.55)	10 (1.86)	χ^2^ = 0.322	0.570	1.207 (0.629–2.316)	0.571
Other pathogens	770 (9.43)	690 (9.05)	80 (14.90)	χ^2^ = 20.094	< 0.001	1.760 (1.370–2.260)	< 0.001
90-day mortality, n(%)	2009 (24.61)	1830 (24.00)	179 (33.33)	χ^2^ = 23.569	< 0.001	1.584 (1.314–1.909)	< 0.001
180-day mortality, n(%)	2171 (26.60)	1969 (25.82)	202 (37.62)	χ^2^ = 35.762	< 0.001	1.732 (1.444–2.078)	< 0.001

*RDW* red cell distribution width, *COPD* chronic obstructive pulmonary disease, *SOFA* septicemia and heart failure, sequential organ failure assessment, *WBC* white blood cell, *A.baumannii* Acinetobacter baumannii, *P.aeruginosa Pseudomonas aeruginosa*, *MRSA* methicillin-resistant *Staphylococcus aureus K.pneumoniae* Klebsiella pneumonia, *MSSA* methicillin-sensitive Staphylo coccus aureus, E. coli *Escherichia coli*, *SAPSII* simplified acute physiology score II

### Comparison of characteristics between VAP group and non-VAP group

After comparing the clinical characteristics of ICU patients in the VAP group and non-VAP group, we found that the age of patients in the VAP group was younger than the non-VAP group (62.29 years vs 64.19 years, t = 2.260, *P* = 0.009), and the proportions of heart failure (32.03% vs 25.37%, χ^2^ = 11.607, *P* < 0.001), septicemia (31.47% vs 17.36%, χ^2^ = 66.828, *P* < 0.001), antibiotic treatment (Z = 18.883, *P* < 0.001), P. eruginosa (*P* < 0.001), MRSA (4.66% vs 2.05%, χ^2^ = 15.760, *P* < 0.001) and other pathogens (14.90% vs 9.05%, χ^2^ = 20.094, *P* < 0.001) were in the VAP group were higher than non-VAP group. The SAPSII score (41.00 vs 38.00, Z = 3.679, *P* < 0.001), SOFA score (7.581 vs 7.096, t = − 3.040, *P* = 0.002), serum lactate (1.80 mmol/L vs 1.60 mmol/L, Z = 2.773, *P* = 0.006), WBC (12.00 10^9^/L vs 11.50 10^9^/L, Z = 2.068, *P* = 0.039) in the VAP group were higher than the non-VAP group. The length of ICU stay (12.69 days vs 2.99 days, Z = 27.865, *P* < 0.001), length of hospital stay (18.79 days vs 8.09 days, Z = 21.875, *P* < 0.001) and length of ventilation (216.47 h vs 20.86 h, Z = 27.780, *P* < 0.001) in the VAP group were higher than in the non-VAP group. The ethnic distribution was different between the VAP group and non-VAP group (χ^2^ = 15.423, *P* = 0.004). the 90-day mortality (33.33% vs 24.00%, χ^2^ = 23.569, *P* < 0.001) and 180-day mortality (37.62% vs 25.82%, χ^2^ = 35.762, *P* < 0.001) in the VAP group were higher than in the non-VAP group. (Table 2).

### The influence of VAP on the prognosis of ICU patients

**Fig. 2:**
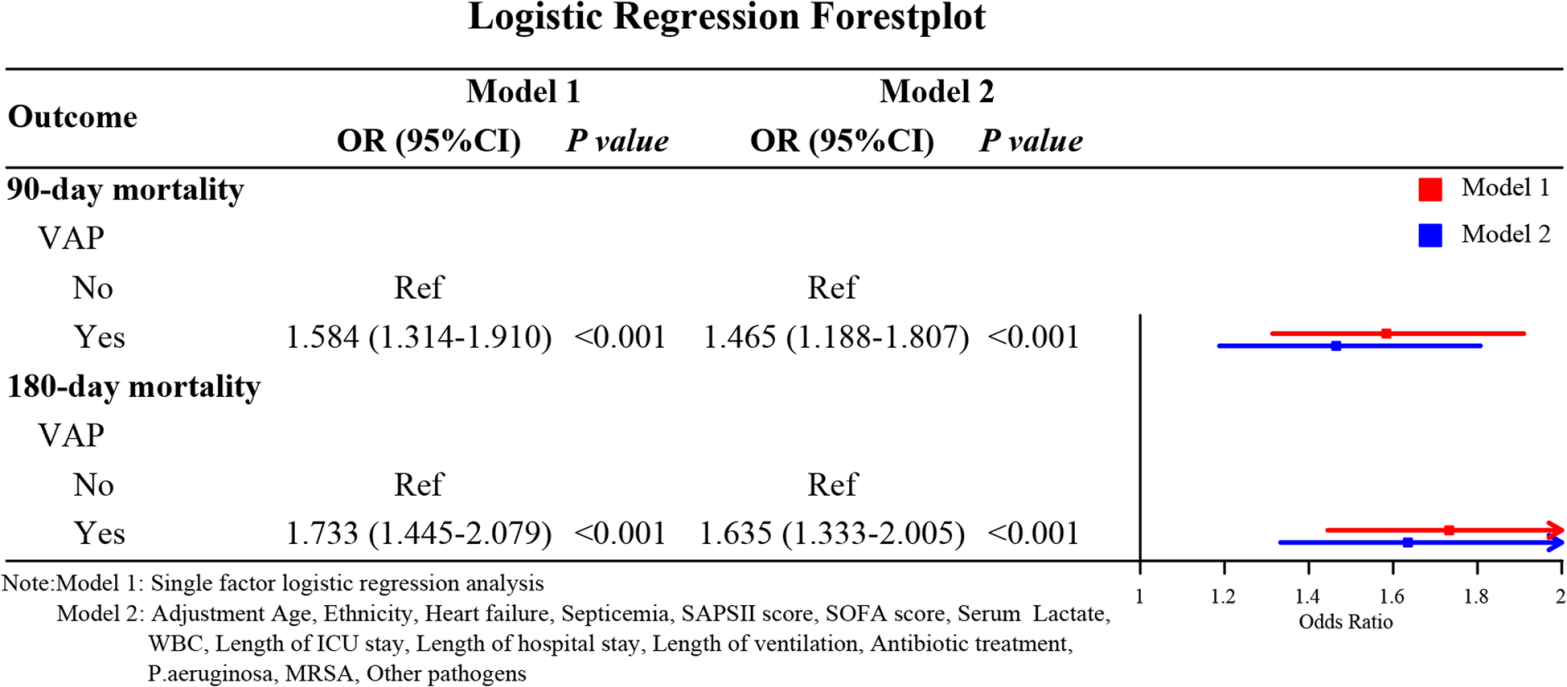
Forest plot of the results based on logistic regression analysis

After adjusting for confounders such as age, ethnicity, serum lactate, septicemia, heart failure, SAPSII score, SOFA score, WBC, length of ICU stay, length of hospital stay, length of ventilation, antibiotic treatment, P.aeruginosa, MRSA, and other pathogens, the results of multivariate logistic regression analysis showed that the 90-day mortality risk of VAP patients was 1465 times (OR = 1.465, 95%CI: 1.188–1.807, *P* < 0.001), and the 180-day mortality risk of VAP patients was 1.635 times (OR = 1.635, 95%CI: 1.333–2.005, *P* < 0.001) compared with those in non-VAP patients (Table 3, Fig. 2).

**Table 3:** The association between VAP and 90/180-day mortality

Outcome	Univariate		Multivariate	
	OR (95%CI)	*P*	OR (95%CI)	*P*
90-day mortality				
VAP				
No	Ref		Ref	
Yes	1.584 (1.314–1.910)	**< 0.001**	1.465 (1.188–1.807)	**< 0.001**
180-day mortality				
VAP				
No	Ref		Ref	
Yes	1.733 (1.445–2.079)	**< 0.001**	1.635 (1.333–2.005)	**< 0.001**

Confounders (Age, Ethnicity, Heart failure, Septicemia, SAPSII score, SOFA score, Serum Lactate, WBC, Length of ICU stay, Length of hospital stay, Length of ventilation, Antibiotic treatment, P.aeruginosa, MRSA, Other pathogens) were adjusted

## Discussion

This study collected the data of 8163 patients receiving MV in ICU from MIMIC III database to investigate the effect of VAP on the prognosis of patients within 90-day and 180-day. From the data we observed that the 90-day and 180-day mortality rates were higher in the VAP group than those in the non-VAP group.

Patients with severe illness are usually admitted into ICU and the mortality of patients in ICU was very high [17]. VAP is one of the common nosocomial infection in ICU [18, 19]. The occurrence of VAP occurs mainly due to the endotracheal tube for delivering MV. Endotracheal tube can produce irritation of the respiratory mucosa, and increase the amount of mucus [20, 21]. Currently, guidelines for preventing VAP were proposed in many studies, including hand washing, elevation of the head of the bed, oral antiseptics and antibiotics, use of endotracheal tubes with subglottic secretion aspiration ports and silver-coated endotracheal tubes, weaning protocols to early extubation, and bundles application, the incidence of VAP is still high [22].

VAP in ICU is associated with increased mortality, and the prognosis of those patients is poor [23]. A retrospective study by Feng et al. reported that in VAP patients, 24.8% were aged > 70 years old and the 30-day mortality was as high as 42.8% [24]. In a study of Vallés et al., the data delineated that VAP is associated with excess mortality and the mortality of VAP patients were higher than that in non-VAP patients (45% vs 27.0%) [25]. Another study indicated that the ICU mortality rate of patients with VAP was 32.5% and hospital mortality rate was 42.5%, which was higher than those in patients without VAP and patients with other ICU–hospital-acquired pneumonias [26]. Kobayashi et al. also reported that the ICU mortality of VAP patients was about 25.0% compared with 13.9% in patients without VAP [27]. The findings of these studies were allied with the results of our study. Herein, we found the 90-day and 180-day mortality rates were higher in the VAP group than in the non-VAP group. The 90-day mortality risk of VAP patients was 1.465 times, and the 180-day mortality risk of VAP patients was 1.635 times than those in patients without VAP. A high risk of mortality of VAP patients in ICU receiving MV indicated that to prevent the occurrence of VAP is of great importance. Factors including mechanical ventilation time, and use of antibiotics affected the occurrence of VAP [28], demonstrating appropriately use of MV and antibiotics was required in ICU patients. Previous studies also indicated that the comprehensive nursing intervention including traditional clinical nursing techniques, various examination measures, and drug intervention, as well as interventions to improve the cognition, psychological state and behavior in patients could prevent the occurrence of VAP, shorten the ventilation time, lower the lung damage and improve the prognosis [29]. Therefore, nurses in ICU should be more strictly trained to take care of these patients.

There were some strengths in our study. Firstly, this was a study with a large scale of sample size based on MIMIC III database, and the sample size was larger than previous studies. Secondly, we adjusted variables with statistical differences including age, ethnicity, heart failure, septicemia, SAPSII score, SOFA score, serum lactate, WBC, length of ICU stay, length of hospital stay, length of ventilation, antibiotic treatment, P.aeruginosa, MRSA, other pathogens between VAP group and non-VAP group, which might have more reliable results. Several limitations existed in this study. Compared with other studies, the variables could be collected from MIMIC III database were limited and incomprehensive, correlated variables including underlying diseases of patients and was not involved in. Additionally, CDC criteria was used to diagnose VAP, which might overestimate the incidence of VAP and this may cause the selection bias in patients. Multi-centers studies with more correlated variables should be conducted to support the conclusions of our study.

## Conclusions

The present study evaluated the 90-day or 180-day mortalities of ICU patients with VAP and the effect of VAP on the risk of mortality in patients. The results observed the poorer prognosis of patients with VAP in ICU than non-VAP patients. The findings of this study might have significant implications for increasing the knowledge of preventing the occurrence of VAP.

## Data Availability

The datasets generated and/or analyzed during the current study are available from the corresponding author on reasonable request.

## Abbreviations

MV: Mechanical ventilation
ICU: intensive care unit
VAP: ventilator-associated pneumonia
MIMIC III: Medical Information Mart for Intensive Care III
RDW: red cell distribution width
SOFA: sequential organ failure assessment
CDC: Centers for Disease Control and Prevention
COPD: chronic obstructive pulmonary disease
WBC: white blood cell
A.baumannii: Acinetobacter baumannii
P.aeruginosa: *Pseudomonas aeruginosa*
MRSA: methicillin-resistant *Staphylococcus aureus*
K.pneumoniae: Klebsiella pneumonia
MSSA: methicillin-sensitive Staphylo coccus aureus
*E. coli*: *Escherichia coli*
SAPSII: simplified acute physiology score II
